# Dioecy, more than monoecy, affects plant spatial genetic structure: the case study of *Ficus*

**DOI:** 10.1002/ece3.739

**Published:** 2013-08-28

**Authors:** Alison G Nazareno, Ana L Alzate-Marin, Rodrigo Augusto S Pereira

**Affiliations:** 1 Federal University of Santa Catarina, UFSCAvenida Ademar Gonzaga, 1346, 88040-000, Florianópolis, Santa Catarina, Brazil; 2 Programa de Pós-graduação em Biologia Comparada, FFCLRP/USPAvenida Bandeirantes, 3900, 14049-900, Ribeirão Preto, Sao Paulo, Brazil; 3 Laboratório de Genética Vegetal, Departamento de Genética, FMRP/USPAvenida Bandeirantes, 3900, 14049-900, Ribeirão Preto, Sao Paulo, Brazil; 4 Departamento de Biologia, FFCLRP/USPAvenida Bandeirantes, 3900, 14049-900, Ribeirão Preto, Sao Paulo, Brazil

**Keywords:** Bayesian clustering, *Ficus citrifolia*, *Ficus eximia*, gene flow, *Sp* statistic

## Abstract

In this analysis, we attempt to understand how monoecy and dioecy drive spatial genetic structure (SGS) in plant populations. For this purpose, plants of the genus *Ficus* were used as a comparative model due to their particular characteristics, including high species diversity, variation in life histories, and sexual systems. One of the main issues we assessed is whether dioecious fig tree populations are more spatially genetically structured than monoecious populations. Using the *Sp* statistic, which allows for quantitative comparisons among different studies, we compared the extent of SGS between monoecious and dioecious *Ficus* species. To broaden our conclusions we used published data on an additional 27 monoecious and dioecious plant species. Furthermore, genetic diversity analyses were performed for two monoecious *Ficus* species using 12 microsatellite markers in order to strengthen our conclusions about SGS. Our results show that dioecy, more than monoecy, significantly contributes to SGS in plant populations. On average, the estimate of *Sp* was six times higher for dioecious *Ficus* species than monoecious *Ficus* species and it was two times higher in dioecious than monoecious plant species. Considering these results, we emphasize that the long-distance pollen dispersal mechanism in monoecious *Ficus* species seems to be the dominant factor in determining weak spatial genetic structure, high levels of genetic diversity, and lack of inbreeding. Although *Ficus* constitute a model species to study SGS, a more general comparison encompassing a wider range of plants is required in order to better understand how sexual systems affect genetic structure.

## Introduction

Reproduction is a driving force in plant evolution that has been a major topic of investigation since the days of Charles Darwin (Darwin 1877; Bawa 1974; Charlesworth 2006). Plant species can be cosexual or unisexual: in cosexual species both male and female functions occur within the same individual (hermaphroditic and monoecious species); whereas in unisexual species, the male and female sexual functions exist in different individuals (dioecious species; Barret 2002). These sexual systems, along with the mode of fertilization or mating system, have driven genome evolution (Charlesworth 1992; Charlesworth and Wright 2001; Wright et al. 2002, 2008; Haudry et al. 2008) and levels of genetic diversity in plant species (Charlesworth and Charlesworth 1995; Hamrick and Godt 1996; Nazareno and Carvalho 2009; Willi and Maattanen 2011).

While mating systems are expected to have a significant effect on genetic diversity (Charlesworth and Wright 2001), they can also have a nonrandom impact on the spatial distribution of genotypes (i.e., spatial genetic structure – SGS) within natural plant populations (Vekemans and Hardy 2004; Hardy et al. 2006; Dick et al. 2008; Lasso et al. 2011). Furthermore, SGS may be influenced by additional ecological and genetic factors, including selection pressures (Ng et al. 2006; Zhou and Chen 2010), genetic drift (Vekemans and Hardy 2004), population density (Vekemans and Hardy 2004; Hardy et al. 2006; Zeng et al. 2012), demography (Hamrick et al. 1993; Epperson and Alvarez Buylla 1997; Jones and Hubbell 2006; Zhou and Chen 2010; Vieira et al. 2012), habitat heterogeneity (Listl and Reisch 2012), landscape features (Born et al. 2008), and the species' life-history traits (Vekemans and Hardy 2004; Luna et al. 2005; Hardy et al. 2006; Dick et al. 2008).

For sessile organisms such as plants, limited gene dispersal is perhaps the most prevalent factor that determines SGS. Several studies on plant species have confirmed Wright's model of isolation by distance (IBD, Wright 1943) which suggests that the strength of SGS is affected by seed and pollen dispersal or restricted gene flow (Hamrick et al. 1993; Nason et al. 1997; Heuertz et al. 2003; Vekemans and Hardy 2004; Luna et al. 2005; Hardy et al. 2006; Epperson 2007; Born et al. 2008; Dick et al. 2008; Oddou-Muratorio et al. 2010; Zhou and Chen 2010; Debout et al. 2011; Kettle et al. 2011). SGS is also affected by random genetic drift, which in turn is dependent on demographic population structure (Wright 1943). Indeed, the theory that SGS is inversely proportionate to species density under Wright's IBD model (Heywood 1991; Vekemans and Hardy 2004) is consistent with the argument that when there is a reduction in population density, pollen dispersal distance increases as a result of an increase in pollinator flight distance (Fenster 1991; Born et al. 2008; Zeng et al. 2012). Likewise, the distance of seed dispersal and the extent of SGS are negatively correlated and this correlation appears to be stronger in plant species with limited seed dispersal than in those with long-distance seed dispersal mechanisms (Hamrick et al. 1993; Loiselle et al. 1995; Hardy et al. 2006; Dick et al. 2008).

Although there is a scarcity of studies demonstrating an association between distance of pollen dispersal and SGS (Dick et al. 2008; Kettle et al. 2011), a reduction in the likelihood of population substructuring is expected for plant species that have long-distance pollen flow. Nevertheless, even when a species with long-distance pollen flow, clusters of closely related individuals can occur due to localized seed dispersal. In this context, tree species with long-distance pollen dispersal constitute an optimal model through which to investigate SGS in plants. Furthermore, assessing the impact of cosexual versus unisexual systems on SGS allows for a comprehensive assessment of evolutionary theories as the level and distribution of genetic diversity within and among populations are governed by the mating system.

The genus *Ficus* Linn. (Moraceae) which includes both monoecious and dioecious species, with diverse ecologies and distinctive functional roles in the ecosystem, is an ideal model to investigate how gene dispersal and sexual systems contribute to SGS. Plants of the *Ficus* genus generally exhibit low-density populations (Nazareno and Carvalho 2009) and are part of a very specialized plant–insect mutualism. Each of the approximately 750 species has a mutualistic relationship with minute pollinating wasps (Weiblen 2002). Despite this universal biological characteristic, *Ficus* species differ widely in their ecological traits. Monoecious *Ficus* species are generally large trees that produce massive fruit crops and disperse their pollen over long distances (Nason et al. 1996; Harrison 2003; Zavodna et al. 2005; Ahmed et al. 2009; Nazareno and Carvalho 2009). On the other hand, dioecious *Ficus* species are generally small-sized trees or shrubs that are spatially aggregated, and present a more limited range of pollen dispersal (Harrison and Rasplus 2006). Moreover, dioecious fig trees tend to fruit more frequently than monoecious fig trees. Due to the ecological traits of dioecious *Ficus* species, Dev et al. (2011) hypothesized that dioecious species may have a stronger SGS than monoecious species. Although this hypothesis is stimulating new debates in relation to SGS, it has not been empirically tested for *Ficus* or for other plant species.

Thus, using the *Sp* statistic (Vekemans and Hardy 2004), which allows for quantitative comparisons across different studies, we compared the extent of SGS between monoecious and dioecious *Ficus* species. To broaden our analysis we compared our results to published data on 18 other genera of monoecious and dioecious plant species.

## Material and Methods

This section is organized in two parts. First, we describe the methods used to characterize the levels of diversity and the SGS for two monoecious *Ficus* species. Second, we present the methodology used in order to effectively compare the SGS estimates across 27 other plant species (13 families, 18 genera).

### Study species

**Figure 1:**
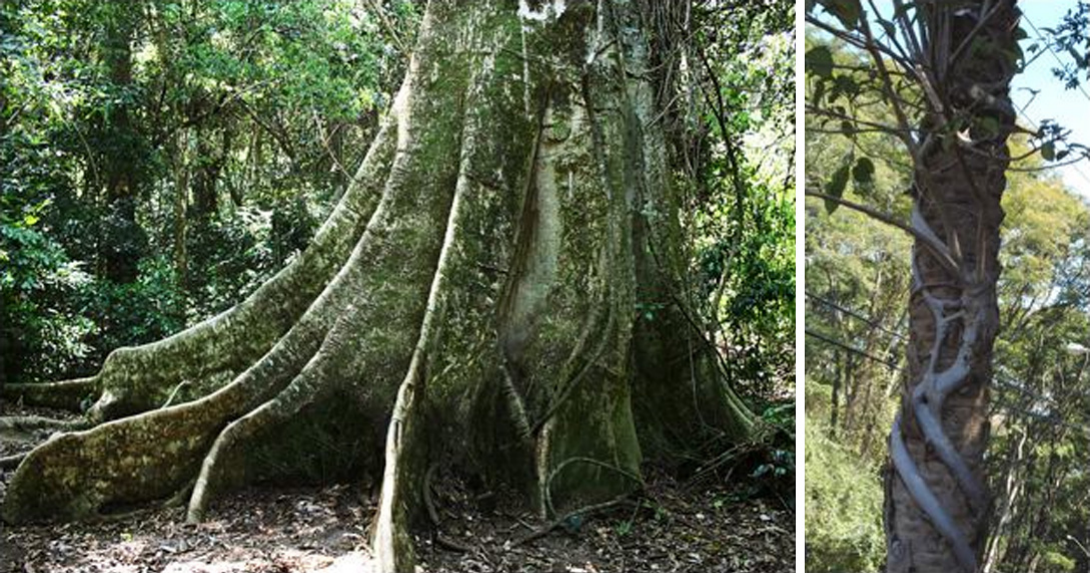
*Ficus citrifolia* P. Miller (right) and *Ficus eximia* Schott (left) at Morro do Diabo State Park, São Paulo State, Southeast Brazil.

*Ficus citrifolia* P. Miller and *Ficus eximia* Schott (Fig. 1) are monoecious Neotropical species belonging to the section *Americana* (Moraceae). Both species occur in the Atlantic Rainforest *sensu lato*. *F. citrifolia* is distributed throughout the Americas, from Florida, to northern Argentina, and *F. eximia* has a range that extends from the Amazon to southern Brazil (Berg and Vilaviccencio 2004). These *Ficus* species are pollinated by species-specific agaonid fig wasps (*Pegoscapus* spp., Hymenoptera: Agaonidae). The fruits of both species are dispersed by bats and generalist birds (Lapate 2009).

Despite the fact that the reproductive biology of *F. citrifolia* and *F. eximia* is essentially the same, these species have distinct ecological strategies. *F. eximia* is a large tree (up to 30 m in height) producing massive fig crops. Its seeds usually germinate on fallen trunks and the plant grows as a freestanding tree in humid and shaded patches of the forest floor. Conversely, *F. citrifolia* is a medium-sized tree (usually less than 10 m in height) and produces smaller fig crops than *F. eximia*. *F. citrifolia* is an efficient colonizer of forest edges and disturbed forest areas. It can grow as a freestanding tree or as a hemiepiphyte when its seeds germinate on the crown of a host tree. Due to these characteristics, *F. eximia* seems to be adapted to long-distance pollen and seed dispersal, whereas *F. citrifolia* seems to be adapted to a more local function.

### Study sites and sampling

Individuals of *F. citrifolia* and *F. eximia* were sampled from two semideciduous Atlantic Forest fragments in the state of São Paulo, southeastern Brazil. The first study site, *Morro do Diabo State Park* (MDSP), consists of a forest fragment of 33,000 hectares located in the western border of the state of São Paulo (22° 32'S, 52° 10'W) and it is the last significant remnant of semideciduous Atlantic Forest in the state. The second site is located at *Caetetus Ecological Station* (CES) which is comprised of a forest fragment of 2,179 hectares located 260 km from MDSP (22° 43'S, 49° 13' W).

**Figure 2:**
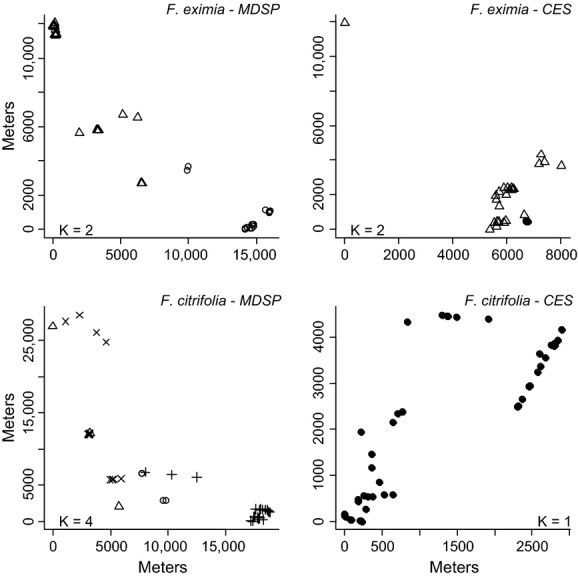
Distribution of the sampled *Ficus eximia* Schott and *Ficus citrifolia* P. Miller individuals in Morro do Diabo State Park (MDSP) and Caetetus Ecological Station (CES) and Bayesian assignments of individuals to subpopulations (K). Each point represents an individual (may be overlapping) and each symbol represents a subpopulation. Note that for *F. eximia*, there was a higher genetic similarity detected inter- than intrapopulation (same symbol in sites MDSP and CES that are separated by 260 km).

Reproductive trees of *F. citrifolia* and *F. eximia* were sampled at both study sites (Fig. 2): for *F. citrifolia,* 82 individuals were sampled from MDSP and 48 from CES; for *F. eximia,* 46 individuals were sampled from MDSP and 28 from CES. Due to the low population densities of both species, individuals were randomly chosen, mapped, and sampled across large areas of each site. From each tree, several leaf samples were collected and stored at −20°C until DNA extraction.

### Genotyping

DNA was extracted from 10 mg of frozen *F. citrifolia* and *F. eximia* leaves using a cetyltrimethyl ammonium bromide (CTAB) extraction method (Alzate-Marin et al. 2009). Twelve microsatellite loci, previously developed for *Ficus insipida* (FINST7, FINSN1) (Vignes et al. 2006), *Ficus racemosa* (FRAC86, FRAC154), and *Ficus rubiginosa* (FRUB29, FRUB38, FRUB61, FRUB391, FRUB415, FRUB416, FRUB422, FRUB436) (Crozier et al. 2007) were screened (for technical details see, Table S1). These microsatellite primers were previously characterized for a subsample of individuals from different populations of *F. citrifolia* and *F. eximia* (Nazareno et al. 2009). Microsatellite polymerase chain reaction (PCR) amplification was conducted in volumes of 10 μL consisting of 0.3 μmol/L of each primer, 1 U *Taq* DNA polymerase, 0.25 mmol/L of each dNTP, 1× MgCl_2_ free of reaction buffer (75 mmol/L Tris-HCl pH 9.0, 50 mmol/L KCl, and 20 mmol/L (NH_4_)_2_SO_4_), 1.5 mmol/L MgCl_2_, and 25 ng of template DNA. Amplified fragments were separated on 10% denaturing polyacrylamide gels in 1× TBE buffer with 8 mol/L of urea followed by staining with silver nitrate. Allele size quantification was estimated using a 10-base pair DNA ladder (Invitrogen*™*, Carlsbad, CA).

### Genetic analysis

The microsatellite data analyses followed a sequential step approach. Due to our sampling strategy, and because of an a priori assignment of individuals to specific populations, biases can be introduced in population genetic inferences (Mank and Avise 2004). Thus, the genetic homogeneity of each ‘population’ was therefore checked using Bayesian clustering. Patterns of genetic diversity and extent of spatial genetic structure (SGS) between *Ficus* species were compared. Finally, indirect gene dispersal distances (σ) were estimated from the population density for populations that showed SGS consistent with the expectations of isolation by distance (IBD) (see Wright 1943).

### Identification of genetic units within populations

In order to detect clusters (i.e., subpopulations) from individual genotypes and spatial information, a Bayesian model was conducted using a Markov chain Monte Carlo (MCMC), as implemented in the R package GENELAND, version 4.0.2 (Guillot 2012). This method operates by minimizing the Hardy–Weinberg and linkage disequilibrium that would result if individuals from different, randomly mating populations were incorrectly grouped into a population. Using this model, the number of clusters is treated as a parameter processed by the MCMC scheme without any approximation, and studies have shown that such an approach may provide a better estimation of the number of clusters than other proposed procedures (Coulon et al. 2006; Guillot et al. 2008). A spatial model was used with correlated allele frequencies as proposed and implemented by Guillot et al. (2008, 2012). As demonstrated by Guillot et al. (2008), the spatial model bases inferences on a prior set of data that are more informative than the uncorrelated frequencies model and hence provides more accurate results. For each *Ficus* species, 100 independent runs were made of 1,000,000 in length, discarding the first 100,000 iterations (burn-in) in postprocessing. The number of subpopulations (*K*) was unknown and hence treated as a simulated variable along with the MCMC simulations (2 ≤ *K* ≤ 10). Null allele frequencies were considered along the clustering algorithm. The number of clusters simulated from the posterior distribution was obtained for both species. Based on the posterior probability of an individual belonging to a certain population, maps for each species and clusters were performed using the R program (R Development Core Team 2009).

### Genetic diversity

Independence among loci was assumed as the 12 microsatellite loci characterized for *F. citrifolia* and *F. eximia* showed locus-independent segregation (Nazareno et al. 2009). Allele frequencies and the following parameters were calculated for both *Ficus* species: allelic richness (*A*), observed heterozygosity (H_*O*_), and expected heterozygosity (*H*_E_) in Hardy–Weinberg equilibrium. Rarefaction approach was used to standardize *A* to the smallest sample size in each comparison. Genetic differentiation (*F*_ST_) for all *F. citrifolia* and *F. eximia* populations was estimated following Weir and Cockerham (1984). These analyses were run using the program FSTAT 2.9.3.2 (Goudet 2002). When applicable, the Kolmogorov–Smirnov test (Zar 1996) was performed to verify if there are genetic differences between the *Ficus* species. The inbreeding index (*F*_IS_) was estimated and its significance (determined by 10,000 permutations across loci) tested using the SPAGeDi program (Hardy and Vekemans 2002). Null allele frequencies were assessed for both populations of each *Ficus* species using the Microchecker software 2.2.0 (van Oosterhout et al. 2004). If significant homozygosity was detected at a given locus, it was dropped and a modified average *F*_IS_ over loci was calculated. Significance was calculated using the jackknife method across all loci. Although it is recommended that *F*_ST_ is corrected for null alleles (Chapuis and Estoup 2007), neither analysis was performed as null alleles have a minimal effect on *F*_ST_ estimates (Yu et al. 2010).

### Spatial Genetic Structure (SGS)

The SGS of both Neotropical *Ficus* species was assessed using the software SPAGeDi (Hardy and Vekemans 2002), following the procedure described by Vekemans and Hardy (2004), based on pairwise kinship coefficients between individuals. We conducted Nason's estimator of kinship coefficient (*F*_*ij*_), as described in Loiselle et al. (1995), as it displays robust statistical properties (Vekemans and Hardy 2004). We identified at least seven distance classes for *F. citrifolia* and *F. eximia* in order to reach a minimum of 100 pairs of individuals per distance class. The average multilocus relationship coefficients per distance class were estimated and their significance per class was tested with 10,000 permutations of multilocus genotypes. To visualize the SGS, we plotted the kinship coefficient against geographical distance. For each *Ficus* population, we tested SGS by assessing the significance of the regression slope (*b*) estimated from the regression between kinship coefficient values and logarithm of the spatial distance between individuals by 10,000 permutations using SPAGeDi (Hardy and Vekemans 2002). This procedure was also applied to each genetic cluster (with a minimum of 45 individuals) detected within populations by the Bayesian model. We did not perform the SGS analysis for *F. eximia* in the CES population because of the low number of sampled individuals (*n* = 28).

From the effective population density *D*_*e*_ (the product of the census density *D* and the *N*_*e*_*/N* ratio), we also estimated the value of the mean parent–offspring distance (σ) for those *Ficus* populations exhibiting a SGS consistent with IBD. In natural plant populations *D*_*e*_ can be estimated as *D*/4 (Hardy et al. 2006). Therefore, the census densities for *F. citrifolia* (CES = 6.59 ind.ha^−1^; MDSP = 2.02 ind.ha^−1^) and for *F. eximia* (CES = 0.24 ind.ha^−1^; MDSP = 0.16 ind.ha^−1^) were multiplied by 0.25.

### Reanalysis of SGS for dioecious and monoecious plants

We compiled published data from 27 plant species (Table 2) belonging to 13 botanical families (Anacardeaceae, Araucariaceae, Burseraceae, Cupressaceae, Dioscariaceae, Ericaceaceae, Fagaceae, Meliaceae, Moraceae, Myristicaceae, Pinaceae, Theaceae, and Sapindaceae) to assess the relationship between SGS and the sexual system. As *Sp* is a synthetic measure of SGS intensity that is less sensitive to the sampling scheme (Vekemans and Hardy 2004; Hardy et al. 2006), we used it to compare the extent of SGS among species and populations. The *Sp* statistic is defined as: *Sp* = −*b*_log_/(1−*F*_(1)_), where *b*_log_ is the regression slope of *F*_*ij*_ on log spatial distance, and *F*_(1)_ is the mean of *F*_*ij*_ between individuals for the first distance class (Vekemans and Hardy 2004).

To compare the levels of SGS between monoecious and dioecious *Ficus* species, we used published *Sp* statistics of four dioecious *Ficus* species: *F. hispida*, *F. exasperata* (Dev et al. 2011), *F. cyrtophylla* (Zhou and Chen 2010), and *F. pumila* (Wang et al. 2009). To assess whether the observed SGS across monoecious and dioecious fig trees constitutes a pattern in flowering plants, we compared our results to published data from 23 plant species (12 monoecious and 11 dioecious plants, see Table 2), representing 13 plant families.

For studies that did not report the *Sp* statistic, we reanalyzed SGS data to either: (1) estimate *Sp* from significant patterns of SGS plotted on spatial autocorrelograms; or (2) deduce *Sp* from other genetic parameters (e.g., Wright's neighborhood size, *Nb* = 1/*Sp*) as proposed by Vekemans and Hardy (2004)).

In order to test whether SGS differed across monoecious and dioecious fig trees, as well as across other plant species, Analysis of Variance (ANOVA) was performed from log *Sp* values; log transforming for *Sp* values was calculated in order to reduce heteroscedasticity.

## Results

### Genetic structure of monoecious *Ficus* species

*Ficus eximia* population showed weaker SGS, with higher genetic similarity on a broad spatial scale (*ca*. 260 km) than *F. citrifolia*. For *F. eximia,* we detected three population groups for the posterior distribution of *K*. Hence, the most appropriate number of clusters for the *F. eximia* populations seems to be three, with two genetic units in each population (Fig. 2). For *F. citrifolia*, we detected five groups for the posterior distribution of *K*. Within the *F. citrifolia* populations, there were four genetic units in the Morro do Diabo State Park (MDSP) and no clear genetic discontinuity in Caetetus Ecological Station (CES) (Fig. 2).

Regardless of the genetic units, we detected high levels of genetic variability in both *Ficus* species in all study populations (Table 1). In a set of 12 microsatellite loci, a total of 91 alleles were detected for *F. citrifolia* and 76 alleles for *F. eximia* species across all populations (Table S1). Private alleles were detected for *F. citrifolia* and *F. eximia* in both MDSP and CES populations. In *F. eximia*, the percentage of private alleles was 14.6% in MDSP and 10.7% in CES. The percentage of private alleles for *F. citrifolia* was 19.5% in MDSP and 2.2% in CES. The average observed number of alleles per locus for *F. citrifolia* was 8.18 (ranging from four to 15) and 6.36 (ranging from two to 12) for *F. eximia* (Table S1). There was no difference in the number of alleles per locus between *Ficus* species (Kolmogorov–Smirnov goodness-of-fit test, *D* = 0.167, *P* > 0.05). Additionally, the species did not differ significantly in terms of genetic diversity. The one-tailed *P*-values after 5000 permutations for the hypothesis that genetic diversity is higher in *F. citrifolia* than *F. eximia* are as follow: allelic richness by rarefaction (*F. citrifolia* = 6.082, *F. eximia* = 5.619, *P* = 0.0646), observed heterozygosity (*F. citrifolia* = 0.559, *F. eximia* = 0.608, *P* = 0.888), and genetic diversity (*F. citrifolia* = 0.664*, F. eximia* = 0.677, *P* = 0.769). Expected and observed heterozygosity and the allele size range for each microsatellite locus in both *Ficus* species are provided in Table S1.

**Table 1:** Population genetics estimates and spatial genetic structure (SGS) parameters for *Ficus citrifolia* P. Miller and *Ficus eximia* Schott in Morro do Diabo State Park (MDSP) and Caetetus Ecological Station (CES) populations, in São Paulo State, Southeast Brazil

Species/Population	n	*A*_*R*_	*H*_*E*_/*H*_*O*_	*F*_IS_	*F*_IS_^1^	*b*_log_ (*R*^2^_log_)	*F*_1_	*Sp*	σ	Ind/ha
*Ficus citrifolia*										
CES	46	6.2	0.687/0.500	0.106*	−0.113*	−0.008298* (0.008)	0.036	0.0086	263	6.59
MDSP	82	7.5	0.667/0.543	0.135*	0.086^ns^	−0.007254* (0.035)	0.035	0.0075	145	2.02
MDSP (Group 1)	49	6.9	0.642/0.563	0.073^ns^	−0.019^ns^	−0.004169* (0.006)	0.019	0.0042	184	2.02
*Ficus eximia*										
CES	28	5.8	0.677/0.601	0.095^ns^	−0.217*					
MDSP	48	6.1	0.687/0.617	0.079^ns^	−0.202*	−0.006101* (0.010)	0.032	0.0063	1221	0.16

*A*_R_, allelic richness by rarefaction; *H*_E_ and *H*_O_, expected and observed heterozygosity, respectively; *F*_IS_, inbreeding index; *F*_IS_^1^, inbreeding index excluding the loci segregating for null alleles; *b*_log_, the regression slope of *F*_*ij*_ on log spatial distance, given with associated determination coefficient *R*^2^; *F*_1_, kinship coefficient between adjacent individuals for the first distance class; *Sp*, intensity of SGS. The mean parent–offspring distance (σ) in meters is also presented. ns (not significant) at *P* > 0.05. *Significant values *P* < 0.05.

On a broad geographical scale, populations of both species showed very low levels of genetic differentiation although they were not significantly different from zero. These results indicate that genetic diversity was distributed mainly within populations: 97.6% in *F. citrifolia* (*F*_ST_ = 0.024, CL_95%_ = 0.0001–0.0341) and 98.6% in *F. eximia* (*F*_ST_ = 0.0136, CL_95%_ = 0.0002–0.0297). Even with the presence of null alleles (Table S1), *F*_IS_ values were not significantly different from zero for all *F. eximia* populations. However, we detected unusual levels of inbreeding among *F. citrifolia* as the overall *F*_IS_ was positive and significantly different from zero for both populations (Table 1). As this level of inbreeding is not expected for an outcrossing, protandrous species that has flower synchrony at an individual level, this pattern of homozygosity was interpreted to be indicative of null alleles (Table S1). When loci with significant null alleles were omitted, the multilocus mean *F*_IS_ showed the expected pattern of no inbreeding (Table 1). As the frequencies of null alleles were very similar throughout the studied populations (Table S2), and because null alleles were accounted for in SPAGeDi, we did not omit loci with null alleles in the SGS analysis.

**Figure 3:**
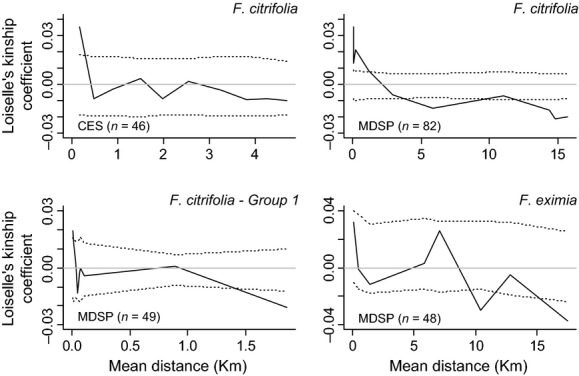
Average Loiselle's kinship coefficient plotted against geographical distance between individuals (solid lines) for *Ficus citrifolia* P. Miller and *Ficus eximia* Schott in Morro do Diabo State Park (MDSP) and Caetetus Ecological Station (CES) populations, in São Paulo State, Southeast Brazil. Dotted lines represent 95% confidence intervals. When the kinship coefficient lies above its 95% confidence limit, individuals are significantly more similar than would be expected through random sampling. As genetic clusters were detected in the MDSP population for *F. citrifolia*, results are also given for Group 1 (49 individuals).

Populations of *F. citrifolia* and *F. eximia* were spatially genetically structured (Fig. 3). Slopes (*b*_log_) of correlograms for all *F. citrifolia* and *F. eximia* populations were significantly different (*P* < 0.01) from the null hypothesis of no SGS (*b*_log_ = 0). The rate of decrease in pairwise kinship coefficients between individuals with the logarithm of the distance, the *Sp* statistic, are similar for the studied monoecious *Ficus* species, with mean values ranging from 0.0062 in *F. eximia* to 0.0081 in *F. citrifolia* (Table 1). The estimates of parent–offspring distances were higher in *F. eximia* than *F. citrifolia* (Table 1).

### SGS across plant species

The comparison of SGS patterns showed that dioecious *Ficus* species have *Sp* values 4.3–11.8 times higher (mean of 6.3) than monoecious species (Tables 2 and 3). Sexual systems (monoecy and dioecy) significantly influenced patterns of SGS across all studied plant species. On average, *Sp* was six times higher in dioecious *Ficus* species than in monoecious *Ficus* species and it was two times higher in dioecious than in monoecious plant species (Table 3). In addition, the effect of sexual system on SGS was still present when we analyzed only plant species studied with microsatellite markers (*n* = 23, *P* = 0.0012, df = 1, *F* = 13.726, one-way ANOVA) and when we analyzed only tree species (*n* = 26, *P* = 0.0027, df = 1, *F* = 10.998, one-way ANOVA).

**Table 2:** *Sp* statistic of spatial genetic structure (SGS) of *Ficus* (Moraceae) and other plant species

Species*	Sexual System	Pollen vector	Seed dispersal	Life form	*F*	*Sp*^npop^	Reference
1 *Ficus citrifolia*^M^	Monoecy	Insect	Animal/gravity	Small tree or hemiepiphyte	−0.113	0.0068^**2**^	This study.
2 *Ficus eximia*^M^	Monoecy	Insect	Animal/gravity	Tree	−0.209	0.0063^**1**^	This study.
3 *Ficus hispida*^M^	Dioecy	Insect	Animal/gravity	Small tree	0.230	0.0350^**1**^	Dev et al. 2011;
4 *Ficus exasperata*^M^	Dioecy	Insect	Animal/gravity	Tree	0.390	0.0311^**1**^	Dev et al. 2011;
5 *Ficus pumila*^M^	Dioecy	Insect	Animal/gravity	Climbing	0.287	0.0742^**1**^	Wang et al. 2009;
6 *Ficus cyrtophylla*^M^	Dioecy	Insect	Animal/gravity	Small tree	na	0.0291^**1**^	Zhou and Chen 2010;
7 *Acer pseudoplatanus*^M^	Monoecy	Insect/wind	Wind/Water	Tree	na	0.0170^**2**^	Pandey et al. 2012;
8 *Carapa guianensis*^M^	Monoecy	Insect	Animal/gravity/water	Tree	0.029	0.0045^**1**^	Cloutier et al. 2007;
9 *Carapa procera*^R^	Monoecy	Insect	Animal	Tree	0.143	0.0280^**1**^	Hardy et al. 2006;
10 *Fagus sylvatica*^M^	Monoecy	Wind	Gravity/animal	Tree	na	0.0219^**3**^	Oddou-Muratorio et al. 2010;
11 *Fagus crenata*^M^	Monoecy	Wind	Gravity/animal	Tree	na	0.0032^**1**^	Oddou-Muratorio et al. 2010;
12 *Larix laricina*^A^	Monoecy	Wind	Wind	Tree	0.024	0.0045^**1**^	Vekemans and Hardy 2004;
13 *Quercus ilex*^M^	Monoecy	Wind	Animal	Tree	−0.026	0.0035^**1**^	Soto et al. 2007;
14 *Quercus lobata*^M^	Monoecy	Wind	Animal	Tree	−0.020	0.0046^**1**^	Dutech et al. 2005;
15 *Quercus petraea*^M^	Monoecy	Wind	Animal/gravity	Tree	0.059	0.0083^**1**^	Vekemans and Hardy 2004;
16 *Quercus robur*^M^	Monoecy	Wind	Animal/gravity	Tree	0.077	0.0030^**1**^	Vekemans and Hardy 2004;
17 Quercus suber^M^	Monoecy	Wind	na	Tree	−0.036	0.0229^**1**^	Soto et al. 2007;
18 *Thuja occidentalis*^M^	Monoecy	Wind	Wind	Tree	0.019	0.0185^**2**^	Pandey and Rajora 2012;
19 *Aucoumea klaineane*^M^	Dioecy	Insect	Wind	Tree	0.088	0.0111^**6**^	Born et al. 2008;
20 *Araucaria angustifolia*^M^	Dioecy	Wind	Animal/gravity	Tree	na	0.0130^**4**^	Stefenon et al. 2008;
21 *Milicia excelsa*^M^	Dioecy	Wind	Animal	Tree	0.077	0.0063^**1**^	Bizoux et al. 2009;
22 *Bagassa guianensis*^M^	Dioecy	Insect	na	Tree	−0.050	0.0388^**1**^	Silva et al. 2008;
23 *Ceratiola ericoides*^A^	Dioecy	Wind	Gravity/Animal	Shrub	na	0.0225^**2**^	Trapnell et al. 2008;
24 *Dioscorea japonica*^M^	Dioecy	Insect	Gravity/Animal	Climbing	0.000	0.0140^**1**^	Mizuki et al. 2010;
25 *Eurya emarginata*^A^	Dioecy	Insect	Gravity	Tree	0.000	0.0246^**1**^	Vekemans and Hardy 2004;
26 *Myracrodruon urundeuva*^M^	Dioecy	Insect	Wind	Tree	-0.153	0.0269^**1**^	Gaiano et al. 2010;
27 *Protium spruceanum*^A^	Dioecy	Insect	Animal/gravity	Tree	−0.143	0.0110^**2**^	Vieira et al. 2012;
28 *Rhus javanica*^M^	Dioecy	Insect	Animal/gravity	Tree	0.000	0.0145^**1**^	Vekemans and Hardy 2004;
29 *Virola michelli*^R^	Dioecy	Insect	Animal	Tree	0.214	0.0150^**1**^	Hardy et al. 2006

Additional biological characteristics and inbreeding coefficient *F* are also shown. For some species (3, 4, 5, 6, 8, and 22), data have been reanalyzed to obtain *Sp* statistic. We report averages of significant *Sp* statistic and inbreeding coefficient *F* across populations for some of the listed species (1, 7, 10, 18, 19, 20, 23, and 27).

* Genetic marker used in the SGS analysis: A, allozymes; M, microsatellites; R, random amplified polymorphic DNA (RAPD); na, data not available in the cited reference; npop, number of populations analyzed.

**Table 3:** Effects of sexual system on *Sp* statistic of *Ficus* (Moraceae) and other plant species (see Table 2)

Effect	*Sp* statistic					

	*Ficus* species		Other plant species		All plant species	
		
	*n*	Mean (SD)	*n*	Mean (SD)	*n*	Mean (SD)
*Sexual system*						
Monoecy	2	0.0067 (0.0018)	12	0.0116 (0.0093)	14	0.0104 (0.0083)
Dioecy	4	0.0424 (0.0214)	11	0.0183 (0.0095)	15	0.0247 (0.0168)
	ANOVA^1^	*P* < 0.001	ANOVA^1^	*P* < 0.05	ANOVA^1^	*P* < 0.001

To test whether statistics of spatial genetic structure (SGS) is related to monoecy and dioecy, statistical analysis was performed by one-way analysis of variance (ANOVA) after log transformation of *Sp* values.

1 Significance level of an one-way analysis of variance; SD is the standard deviation.

## Discussion

Investigations of the spatial genetic structure in plant populations provide an initial indication of how microevolutionary forces, such as gene flow, influence the distribution of genetic diversity. For plant species that reproduce sexually, gene flow determines the extent to which genes are narrowly or more broadly dispersed (Wright 1943; Loiselle et al. 1995). Although many studies have assessed the SGS for specific plant species in a single or a limited number of populations, comparative studies across taxa and populations are still scarce (Vekemans and Hardy 2004; Hardy et al. 2006; Dick et al. 2008; Harata et al. 2012). In our study, we attempt to determine how monoecy and dioecy drive SGS in plant populations. For this purpose, we used plants of the genus *Ficus* as a comparative model due to their specific characteristics, including high species diversity with different life histories and the presence of both monoecy and dioecy. One of the main issues assessed was whether dioecious fig trees are more genetically structured than monoecious ones. This pattern seems to be related to the ecological traits of monoecious and dioecious fig species. Monoecious figs generally have low population densities and are represented by canopy emergent trees that show asynchronous flowering among individuals but massive fig crops within individuals. On the other hand, dioecious *Ficus* species are generally aggregated understory plants that flower more frequently, showing more asynchronous crops within individuals (Harrison and Shanahan 2005). Therefore, the higher local densities and more frequent flowering of dioecious fig species seem to reduce the evolutionary pressure for long-distance pollen dispersal and increase the probability of nonrandom genetic distribution within populations.

Our results demonstrate that sexual systems (particularly dioecy) can significantly affect SGS in the *Ficus* genus and across plant species (Table 3). As SGS within species can vary by orders of magnitude (Jump et al. 2012), a more general comparison encompassing a wider range of plants and populations is required in order to strengthen our understanding of how sexual systems affect genetic structure. Irrespective of the sexual system, a combination of biological characteristics – including mating system, life form, population density, and pollen and seed dispersal – can also contribute to SGS in plants (Vekemans and Hardy 2004; Hardy et al. 2006). Vekemans and Hardy (2004) demonstrated that mating systems significantly affect SGS in the plant species that reproduce through selfing are on average of 10 times more spatially genetically structured than outcrossing species. However, a mating system per se does not prevent SGS. In the absence of selection, the SGS of a plant species will be affected only if gene dispersal is limited. As pointed out previously (Hamrick et al. 1993; Loiselle et al. 1995; Hardy et al. 2006; Dick et al. 2008; Choo et al. 2012; Harata et al. 2012), the strength of local SGS can be broadly predicted from the efficiency of seed dispersal. From this point of view, our results suggest that dioecious plant species, which on average were two times more structured than monoecious species (Table 3), have narrower ranges of gene dispersal. This conclusion is contrary to previous studies on animal-pollinated species (Yu et al. 2010; Zhou and Chen 2010) and to Fromhage and Kokko's model (Fromhage and Kokko 2010) which predicts that dioecy should evolve if seeds and pollen are widely dispersed. However, our argument is supported by several factors, including: the occurrence of inbreeding (i.e., biparental inbreeding) in several dioecious plant species (Giles and Goudet 1997; Segarra-Moragues et al. 2005; Hardy et al. 2006; Miller and Schaal 2006; Born et al. 2008; Bizoux et al. 2009; Wang et al. 2009; Dev et al. 2011; Ferreira et al. 2012); the fact that dioecious species are generally associated with unspecialized pollination systems – for example, wind-, water-pollinated, or generalist pollinators (Barret 2002); and dioecious species generally present low pollen dispersal distances. For instance, Ferreira et al. (2012) suggested that the high inbreeding levels observed in *Araucaria angustifolia*, a dioecious wind-pollinated tree species, are due to the mating of close relatives as a result of near-neighbor pollination and seed dispersal close to the seed trees.

A reduction in SGS is also expected in dioecious species if the limitations that are inherent in their populations are present. For instance, if there is an unbalanced sex ratio, dioecious plants can experience limited gene dispersal and loss of genetic variability due to increased genetic drift (Allendorf and Luikart 2007; Vandepitte et al. 2010). In dioecious species, when only females produce seeds, offspring will be more spatially clumped and will experience more local resource competition than when cosexuals produce seeds (Heilbuth et al. 2001). In addition, Vandepitte et al. (2010) reported that gender proportion is strongly associated with genetic diversity in populations of *Mercurialis perennis*, a dioecious plant species. From another point of view, van Drunen and Dorken (2012) reported that distances between dioecious *Cirsium arvense* females and males limit pollen dispersal more strongly than the overall frequency of males within populations.

We emphasize that the long-distance pollen dispersal mechanism in monoecious *Ficus* species seems to be the dominant factor in determining high levels of genetic diversity, lack of inbreeding, and weak spatial genetic structure. Likewise, for monoecious *Ficus* species, the need to sustain populations of pollinator wasps means that ripe figs can be found year round, attracting and sustaining frugivores throughout the year (Shanahan et al. 2001). This, together with the ability of some vertebrates (e.g., fruit bats) to disperse fig seeds hundreds of kilometers (Shilton et al. 1999), can also weaken SGS, reduce biparental inbreeding, and favor high levels of genetic diversity. Similar to previous genetic analyses of *Ficus* (Nason et al. 1998; Nazareno and Carvalho 2009; Yu et al. 2010; Dev et al. 2011), we found high genetic diversity values in both monoecious species assessed in this study (*F. citrifolia* and *F. eximia*). This recurring pattern in *Ficus* species can be attributed to both the mating system and mutualism with species-specific fig wasps. Specialized pollinators of low-density species, such as the wasps that pollinate fig trees, have evolved the ability to find conspecific adults located at great distances (McKey 1989; Nason et al. 1998; Harrison 2003; Zavodna et al. 2005; Ahmed et al. 2009; Nazareno and Carvalho 2009; Yu et al. 2010). Indeed, molecular data suggest that a pollinating wasp can travel up to 160 km to successfully pollinate a fig tree (Ahmed et al. 2009). This exceptionally long-distance dispersal is achieved through a wind-mediated dispersal mechanism (Nason et al. 1998; Compton et al. 2000; Harrison 2003). Winds blowing above the forest canopy aid in carrying these wasps far enough distances to allow them to reach their hosts. As fig wasps detect the species-specific volatiles produced by their host fig trees (Grison-Pigé et al. 2002), they actively fly in the direction of their host to pollinate the receptive tree. Thus, fig trees have evolved a remarkable mechanism for pollen dispersal, using wind and active chemotaxis by the wasps to overcome the constraint of low population density and achieve sexual reproduction.

Our landscape-level results and previous studies (Nason et al. 1998; Harrison 2003; Zavodna et al. 2005; Saddoud et al. 2007; Ahmed et al. 2009; Nazareno and Carvalho 2009; Yu et al. 2010) suggest that extensive gene flow via pollen dispersal may buffer the effect of genetic drift and moderate levels of microenvironmental selection. As a consequence, fig and pollinating fig wasp populations can cover wide geographical areas (Zavodna et al. 2005; Ahmed et al. 2009; Yu et al. 2010). For instance, the movement of *F. racemosa* pollinator populations has resulted in low levels of genetic differentiation among *F. racemosa* populations over continental Southeast Asia (Kobmoo et al. 2010). This extensive movement across landscapes was also found for other fig wasps (Molbo et al. 2004; Zavodna et al. 2005; Lin et al. 2008), indicating that the delimitation of a population of mating fig trees is not straightforward. Thus, even isolated individuals or isolated populations may be important for the maintenance of genetic diversity of the fig species and pollinator populations. Calculations of the potential for fig wasp dispersion show that a wind blowing with the annual average speed of ∼20 km/h across the studied landscape (Amarante et al. 2001) travels about 250 km in 12 h. This indicates that pollen flow between the *Ficus* populations assessed in our study is feasible, thus validating the very low level of nuclear differentiation (*F*_ST_) observed in both species, as well as the high genetic similarity on a wide spatial scale inferred by Bayesian analysis for *F. eximia* populations (∼260 km). Low nuclear differentiation among populations has also been reported for other *Ficus* species on a broad geographical scale (Saddoud et al. 2007; Yu et al. 2010).

Additional factors that explain the high levels of genetic diversity in monoecious *Ficus* species can be ascribed to weak spatial genetic structure (SGS) detected for both monoecious *F. citrifolia* and *F. eximia* species. As pointed out by Heywood (1991), SGS may be an important driver in the maintenance of plant species genetic variation. Indeed, Willi and Maattanen (2011) found that SGS caused by limited gene flow was positively associated with population genetic variation in *Arabidopsis lyrata* populations. Furthermore, demographic thinning, such as density-dependent competition, can also cause limited or weak SGS. Zhou and Chen (2010) demonstrated that about two thirds of seeds from the dioecious *Ficus cyrtophilla*, which grows mostly in the understory, are locally dispersed. However, they detected a reduction in SGS as the species transitioned from seedlings to saplings and adults, suggesting that demographic selection during recruitment affects SGS. In our study, a weak SGS was observed over a broad spatial scale. Although the mean distance between parent and offspring was higher than that reported for dioecious *Ficus* species (Dev et al. 2011), the low estimates of distance between parent and offspring were especially noteworthy given the capacity for long-distance gene dispersal in monoecious *Ficus* species. However, our results can be attributed to limited seed dispersion, as pollen can be dispersed over very long distances (Ahmed et al. 2009).

Although limited seed dispersal involving animal vectors is generally attributed to the dispersers' foraging behavior, the reduction or loss of habitats, which has occurred in the recent past in the studied *Ficus* populations, may bring about consequences that affect seed dispersal, such as a decline in disperser populations (Lapate 2009). Lower rates of seed dispersal and changes in regenerative potential due to a reduction in fauna populations have been reported for *Ficus* (Kirika et al. 2008) and for other plant species (Letourneau et al. 2004). While we found no inbreeding for monoecious *Ficus* species in this study, similar to the results reported by Nazareno and Carvalho (2009), a decrease in seed establishment as a consequence of loss of fauna could lead to increases in SGS within populations. In a recent study, Yu et al. (2010) reported that ecological predictions of long-distance dispersal of fig seeds is inconsistent with the high level of *cp*DNA differentiation, suggesting that local inbreeding in combination with fine-scale intrapopulation genetic structure is a result of localized seed dispersal.

In summary, this study demonstrates that sexual systems are an important factor affecting SGS in plant species with diverse life-history traits. Notably, dioecious *Ficus* species were on average six times more spatially structured than monoecious *Ficus* species. While our study provides interesting new insight into the role of sexual systems in SGS, a relatively low number of comparative species were used due to a lack of studies reporting SGS for monoecious and dioecious plant species. Clearly, future studies are necessary that compare patterns of spatial genetic structure across a wide range of taxa. Furthermore, given that about half of the *Ficus* species are dioecious (Harrison and Shanahan 2005), we believe that it is critical to sample as many species as possible in order to understand the evolutionary processes that drive SGS, especially for those species that are heavily dependent on specialized pollinators. This is especially a concern in light of global climate change. Long-term studies have provided insights into the effects of climate change on the reproductive biology of plants (Boggs and Inouye 2012), indicating that climate change can disrupt the timing between the flowering and pollinating period, thus significantly altering the intimate relationships that exist in coevolved systems. As a consequence, we can expect an increase in SGS and inbreeding, a decrease in genetic variability, and the possible (local) extinction of plants and their associated taxa.

## Data Accessibility

Null allele frequencies and technical features of microsatellite markers: DRYAD entry doi: 10.5061/dryad.r8f6r.

## Supporting Information

**Table S1.** Technical features of microsatellite primers and the diversity parameters for *Ficus citrifolia* P. Miller and *Ficus eximia* Schott populations, in São Paulo State, Southeast Brazil. *T*_a_, annealing temperature (°C) for *F. citrifolia* (1) and *F. eximia* (2); R, allele size range in base pairs (bp); *nA*, observed number of alleles; *H*_E_ and *H*_O_, expected and observed heterozygosity, respectively; *f*, fixation index calculated as 1 – *H*_O_/*H*_E_.

**Table S2.** Estimates of null allele frequencies for *Ficus citrifolia* P. Miller and *Ficus eximia* Schott in Morro do Diabo State Park (MDSP) and Caetetus Ecological Station (CES) populations, in São Paulo State, Southeast Brazil. *mo*, monomorphic locus; *presence of null alleles based on Oosterhout's method.
