# Supplementary material for: Dioecy, more than monoecy, affects plant spatial genetic structure: the case study of Ficus
Source: Ecol Evol. 2013 Aug 28;3(10):3495–508. doi: 10.1002/ece3.739 (PMC3797494; doi:10.1002/ece3.739)
Supplement: Supplementary file 1 [file ece30003-3495-SD1.docx]

Table S1. Technical features of microsatellite primers and the diversity parameters for *Ficus citrifolia* P. Miller and *Ficus eximia* Schott populations, in São Paulo State, Southeast Brazil. *T*_a_, annealing temperature (ºC) for *F. citrifolia* (1) and *F. eximia* (2); R, allele size range in base pairs (bp); *nA*, observed number of alleles; *H*_E_ and *H*_O_, expected and observed heterozygosity, respectively; *f*, fixation index calculated as 1 – *H*_O_/*H*_E_.

|  |  |  | *Ficus citrifolia* | | | | | *Ficus eximia* | | | | |
| --- | --- | --- | --- | --- | --- | --- | --- | --- | --- | --- | --- | --- |
| Primers | Repeat motif | *T*_a(1)_/*T*_a(2)_ | R (bp) | *nA* | *H*_E_ | *H*_O_ | *f* | R (bp) | *nA* | *H*_E_ | *H*_O_ | *f* |
| FinsT7 ^(a)^ | (TA)_2_(CA)_10_(TA)_7_…(TG)_2_ | 50/49 | 172-184 | 05 | 0.286 | 0.159 | 0.443 | 178 | 01 | -- | -- | -- |
| FinsN1 ^(a)^ | (TA)_11_(TG)_16_ | 46/50 | 150-160 | 04 | 0.688 | 0.957 | -0.393 | 158-164 | 04 | 0.653 | 0.928 | -0.424 |
| Frac86 ^(b)^ | (TC)_13_(CA)_10_ | 46/50 | 166-196 | 15 | 0.910 | 0.552 | 0.395 | 162-178 | 08 | 0.825 | 0.250 | 0.699 |
| Frac154 ^(b)^ | (AC)_13_ | 48/48 | 140 | 01 | -- | -- | -- | 144-160 | 06 | 0.786 | 1.000 | -0.274 |
| Frub29 ^(b)^ | (AG)_24_ | 48/50 | 186-228 | 08 | 0.792 | 0.672 | 0.151 | 195-255 | 12 | 0.868 | 0.695 | 0.200 |
| Frub38 ^(b)^ | (AG)_8_…(GA)_13_ | 50/49 | 190-220 | 07 | 0.773 | 0.667 | 0.138 | 197-227 | 07 | 0.754 | 0.323 | 0.572 |
| Frub61 ^(b)^ | (TC)_24_ | 50/50 | 134-178 | 13 | 0.814 | 0.541 | 0.336 | 120-166 | 10 | 0.864 | 0.597 | 0.310 |
| Frub391 ^(b)^ | (TG)_19_ | 49/50 | 136-158 | 11 | 0.841 | 0.487 | 0.421 | 140-156 | 05 | 0.767 | 0.951 | -0.242 |
| Frub415 ^(b)^ | (AC)_10_ | 50/48 | 132-156 | 04 | 0.532 | 0.570 | -0.072 | 158-164 | 02 | 0.413 | 0.380 | 0.081 |
| Frub416 ^(b)^ | (CA)_14_(CA)_8_ | 49/49 | 212-232 | 06 | 0.779 | 1.000 | -0.284 | 215-230 | 04 | 0.649 | 0.893 | -0.379 |
| Frub422 ^(b)^ | (AC)_18_ | 49/49 | 156-176 | 08 | 0.801 | 0.517 | 0.355 | 156-170 | 08 | 0.762 | 0.710 | 0.069 |
| Frub436 ^(b)^ | (AC)_22_ | 48/48 | 134-178 | 09 | 0.833 | 0.578 | 0.307 | 148-188 | 09 | 0.825 | 0.562 | 0.320 |
| Total | | | | 91 |  |  |  | Total | 76 |  |  |  |

^a^, primers from Vignes et al. 2006; ^b^, primers from Crozier et al. 2007.
