# Supplementary material for: Dioecy, more than monoecy, affects plant spatial genetic structure: the case study of Ficus
Source: Ecol Evol. 2013 Aug 28;3(10):3495–508. doi: 10.1002/ece3.739 (PMC3797494; doi:10.1002/ece3.739)
Supplement: Supplementary file 2 [file ece30003-3495-SD2.docx]

Table S2. Estimates of null allele frequencies for *Ficus citrifolia* P. Miller and *Ficus eximia* Schott in Morro do Diabo State Park (MDSP) and Caetetus Ecological Station (CES) populations, in São Paulo State, Southeast Brazil. *mo*, monomorphic locus; *, presence of null alleles based on Oosterhout’s method.

|  | *Ficus citrifolia* | | *Ficus eximia* | |
| --- | --- | --- | --- | --- |
| Locus | MDSP | CES | MDSP | CES |
| FinsT7 | 0.1609* | 0.1591* | *mo* | *mo* |
| FinsN1 | -0.2417 | -0.2396 | -0.2267 | -0.4799 |
| Frac86 | 0.2317* | 0.0917* | 0.3078* | 0.3416* |
| Frac154 | *mo* | *mo* | -0.1380 | -0.1859 |
| Frub29 | 0.0917* | 0.0446 | 0.0594* | 0.1656* |
| Frub38 | 0.0714* | 0.0496 | 0.2850* | 0.2549* |
| Frub61 | 0.1774* | 0.1461* | 0.1340* | 0.1749* |
| Frub391 | 0.2273* | 0.1574* | -0.1469 | -0.1692 |
| Frub415 | -0.0532 | -0.0265 | -0.0027 | 0.0992 |
| Frub416 | -0.1551 | -0.1786 | -0.3712 | -0.1842 |
| Frub422 | 0.1648* | 0.0834* | 0.0531 | -0.0257 |
| Frub436 | 0.1536* | 0.1445* | 0.1626* | 0.1378* |
